# Skull bone marrow and skull meninges channels: redefining the landscape of central nervous system immune surveillance

**DOI:** 10.1038/s41419-025-07336-2

**Published:** 2025-01-29

**Authors:** Liang Liu, Xian Zhang, Yan Chai, Jianning Zhang, Quanjun Deng, Xin Chen

**Affiliations:** 1 Department of Neurosurgery, Tianjin Medical University General Hospital, Tianjin, P.R. China; 2 Tianjin Neurological Institute, Key Laboratory of Post-Trauma Neuro-Repair and Regeneration in Central Nervous System, Ministry of Education, Tianjin Key Laboratory of Injuries, Variations and Regeneration of Nervous System, Tianjin, P.R. China

**Keywords:** Neuroimmunology, Immune system

## Abstract

The understanding of neuroimmune function has evolved from concepts of immune privilege and protection to a new stage of immune interaction. The discovery of skull meninges channels (SMCs) has opened new avenues for understanding central nervous system (CNS) immunity. Here, we characterize skull bone marrow and SMCs by detailing the anatomical structures adjacent to the skull, the differences between skull and peripheral bone marrow, mainstream animal processing methods, and the role of skull bone marrow in monitoring various CNS diseases. Additionally, we highlight several unresolved issues based on current research findings, aiming to guide future research directions.

## Facts

- Short-lived leukocytes play a crucial role in responding to injury and infection.
- Bone marrow is a key immune organ.
- Hematopoietic stem cell differentiation exhibits lineage bias due to variations in the bone marrow microenvironment and the signals received.
- A bidirectional channel connects the skull bone marrow and meninges.

## Open questions

- How does the CNS balance the relationship between skull bone marrow immunity and peripheral immunity in healthy or pathological conditions?
- Do the density and diameter of SMCs vary in different parts of the skull, leading to functional differences? Is this variation related to the hematopoietic ability of niches in different parts of the skull bone marrow? Are immune cells affected by the heterogeneity of SMCs during migration?
- Cerebrospinal fluid (CSF) can induce peripheral bone marrow hematopoiesis through lymphatic drainage or directly enter the skull bone marrow to stimulate local hematopoiesis through SMCs. What is the proportion of each pathway?
- The hematopoietic function of the skull bone marrow is permanent, but SMCs are bone channels similar to blood vessels. Will they deteriorate with age, affecting the signal reception and transport of cells in the skull bone marrow? Is this related to the occurrence and development of neurodegenerative diseases?
- Is the occurrence of long-term complications in trauma patients related to damage to skull bone marrow niches and SMCs?
- Can the immune cells generated by the skull bone marrow niche be transported to the circulating blood to regulate peripheral organ immunity? How important is this?
- Despite the availability of various methods for studying cells from different bone marrow sources, significant limitations persist. Developing more precise and suitable techniques is essential to minimize their impact on experimental outcomes.

## Introduction

Inflammation is a critical defensive response induced by immune cells. However, excessive immune cells, including neutrophils, monocytes, and macrophages, recruited to inflammatory sites instead of helping and made things worse. After acute brain injury, the circulating immune cells increase greatly due to the destruction of the blood–brain barrier (BBB); the circulating immune cells gradually penetrate the brain tissue and peak in a few days, driving neuroinflammation and neurological deterioration [1–7]. Given the rapid response and short lifespan of immune cells, much research has focused on bone marrow, the site where it is produced [8, 9]. However, it remains unclear whether damage at different sites uniformly increases the activity of the entire hematopoietic system.

**Fig. 1:**
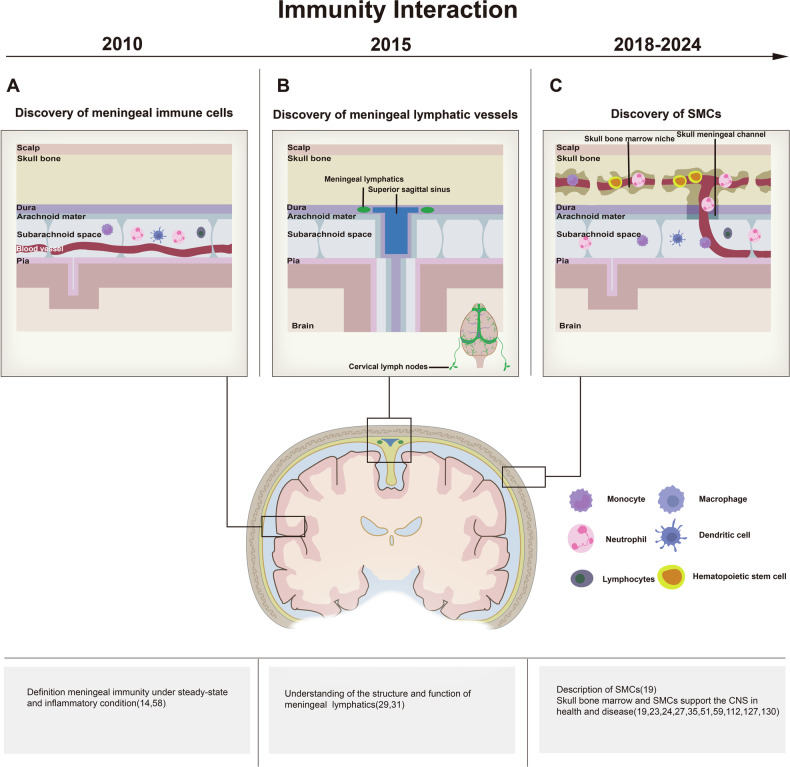
Timeline understanding of neuroimmune interactions. Since the neuroimmune system entered the immune interaction stage, it has undergone three milestone phases. The following three figures illustrate these phases: **A** The discovery of immune cells in the dura mater has reshaped our understanding of meningeal immunity and demonstrated its crucial role in guiding and coordinating CNS immunity. This discovery marked the beginning of the neuroimmune system’s transition into the immune interaction stage. **B** The identification of functional lymphatic vessels in the dura mater sinus challenged the traditional view that the CNS lacks lymphatic vessels. This finding has provided new insights into the pathways for immune cell and metabolic waste clearance within the CNS and has shed light on the development of neurodegenerative diseases. **C** The discovery of SMCs has advanced the understanding of neuroimmune interactions to a new level. It has highlighted the significance of bone marrow hematopoiesis in the skull and refined the analysis of the origin, distribution, function, and heterogeneity of immune cells in both the meninges and brain parenchyma under homeostatic and various pathological conditions.

Recent research has transformed our understanding of brain immunity, shifting from the traditional view of the brain as an immune-privileged organ to recognizing its complex physical and functional connections with the immune system [10]. Historically, this immune-privileged status was attributed to the presence of the BBB, the prolonged survival of transplanted tissues in the brain, and the presumed absence of a lymphatic system [11, 12]. However, the discovery of immune cells in the meninges has demonstrated that the brain is not isolated but actively interacts with the immune system through multiple pathways. Immune cells occupy specialized niches at the brain’s boundaries—such as the meninges, choroid plexus, and perivascular spaces—where they play essential roles in repair, support, and immune surveillance, marking the onset of CNS-immune interactions [10, 13–16] (Fig. 1).

The brain’s immune system interacts with immune cells via several specialized structures. The meninges host various immune cells, including T cells, macrophages, and dendritic cells, which regulate brain function remotely by releasing cytokines [16–19]. Additionally, the meninges connect to cervical lymph nodes through brain lymphatic vessels, facilitating waste clearance and immune surveillance [20, 21]. The choroid plexus, functioning as the blood-cerebrospinal fluid barrier (BCSFB), not only regulates CSF composition but also serves as a gateway for immune cells entering the CNS [22]. Perivascular spaces contribute to the clearance of metabolic waste and antigens via the glymphatic system, which regulates CSF flow and CNS perfusion [23, 24] Furthermore, the skull bone marrow is connected to the meninges via SMCs, enabling CSF to transmit signaling molecules that regulate the maturation and differentiation of immune cells. These immune cells, including myeloid cells and B cells, migrate to the meningeal niche, supporting immune surveillance and rapid immune responses in the brain [25–27]. This discovery challenges previous paradigms of CNS immunity. For example, during CNS infection or inflammation, immune cells such as neutrophils from skull bone marrow can rapidly migrate through these channels to the dura mater, contributing to localized immune responses [25]. Moreover, under certain pathological conditions, skull bone marrow-derived immune cells may exhibit distinct phenotypes and functions compared to those originating from peripheral sources [28–30]. This undoubtedly emphasizes the crucial role of skull bone marrow in the immune regulation of the CNS.

This review provides a comprehensive summary of current knowledge regarding skull bone marrow and its SMCs, highlighting their unique roles in neurological diseases. Additionally, it outlines promising directions for future research into CNS diseases driven by dysregulation of the brain-immune network.

## Anatomical relationships between skull bone marrow and adjacent structures

Specific immune responses of the CNS due to the unique anatomical features [31, 32]. The meninges, comprising the outermost dura mater, intermediate arachnoid membrane, and innermost pia mater, are connected to the skull and brain parenchyma. They contain a variety of immune cells that monitor the CNS microenvironment through the flow of CSF [17, 33, 34].

The dura mater of the skull contains lymphatic vessels that drain CSF, carrying waste products from the brain to peripheral circulation [20, 21, 35]. Geir and Per, using magnetic resonance imaging (MRI) and CSF tracers, found that the parasagittal meninges may act as a bridge for molecular exchange between brain tissue, mediated by CSF, and the lymphatic vessels of the dura mater [20, 21, 36, 37].

**Fig. 2:**
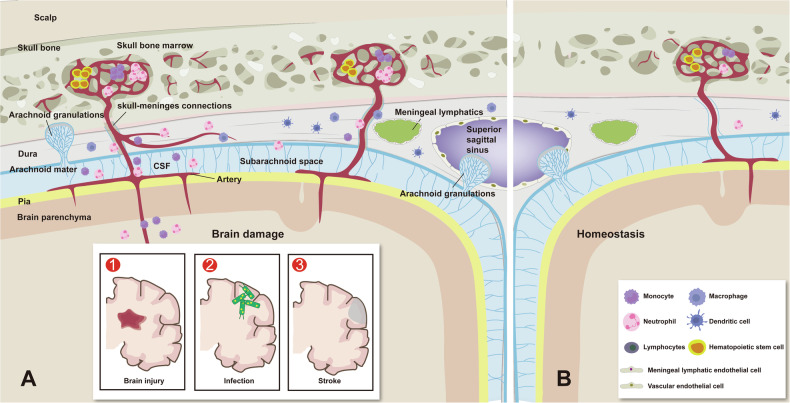
Dynamic changes of skull bone marrow and SMCs in steady-state and various pathological states. The skull bone marrow is the closest to the CNS and is connected to the meninges via specialized structures known as SMCs. These SMCs are bony structures that contain blood vessels integrated with the blood sinuses in the bone marrow and perivascular spaces. They facilitate the transport of immune cells produced in the skull bone marrow to the dura mater through these blood vessels, while also utilizing the perivascular spaces to receive and transmit signals from CSF bidirectionally. **A** Under pathological conditions, CSF can transport various stimulating factors into the skull bone marrow, thereby promoting hematopoiesis and the production of disease-specific immune cells, which subsequently migrate to the dura mater through the SMCs and eventually to the brain parenchyma. **B** In a steady state, the skull bone marrow continues to exhibit hematopoietic activity, supplying immune cells to the dura mater to support its normal immune function.

SMCs are newly discovered physical structures that connect the skull bone marrow and meninges [25], allowing crosstalk between the CSF and skull bone marrow [33] SMCs are composed of a blood vessel fused with a sinusoidal vessel and its surrounding spaces. Hematopoietic stem cells reside in niches around the sinusoids, while different types of progenitor cells are distributed across distinct regions surrounding the sinusoids [38]. Under healthy conditions, the skull bone marrow maintains hematopoietic activity to support normal CNS immune function, but SMCs deliver immune cells to the dura mater at a low frequency. In contrast, under pathological conditions such as infection [33], stroke [25], CSF can transport various stimulatory factors through SMCs to the skull bone marrow. These factors stimulate hematopoiesis, leading to the production of specific immune cells. These immune cells then migrate to the dura mater at a high frequency and eventually infiltrate the brain parenchyma, triggering immune-inflammatory responses (Fig. 2).

Fanny and colleagues found that SMCs are CD31^+^ osseous channels [25]. Additionally, there is a layer of fibroblasts in SMCs that can function as antigen-presenting cells, indicating that the skull bone marrow initiates immune functions when CSF passes through SMCs [39, 40]. Fadi E and colleagues used high-resolution X-ray computed tomography to scan the skull and found that SMCs are densely distributed in the frontal, parietal, and occipital lobes [33]. Intriguingly, the SMCs in different regions of the skull also showed heterogeneity. The frontal and parietal skull channels are shorter and narrower compared to the occipital in mice, and the density of frontal and occipital skull channels is higher than that of the parietal region [33] (Table 1).

**Table 1:** Details of SMCs in different skull bones of mice.

Skull	The density of SMCs (mm^2^)	The length of SMCs (μm)	The diameter of SMCs (μm)
Frontal bone	12–14	83–88	18–23
Parietal bone	2–4	85–90	18–23
Occipital bone	11–13	100–110	20–25

Humans have similar channels connecting the bone marrow and meninges of the skull, but the anatomical structure of human SMCs is different from that of mice.

Compared to mice, human skull bone marrow exhibits a more complex anatomical structure, characterized by wider and more prominent bone marrow cavities, as well as more extensive connections with the meninges [39]. Additionally, human SMCs are more structurally complex than those in mice, with channel widths ranging from 40 to 90 µm, and some exceeding 150 µm. Moreover, human SMCs typically penetrate the dura mater, connect to arachnoid granulations, and may participate in CSF flow and sampling. Furthermore, human SMC channels may contain fat, a feature absent in mouse SMCs, potentially serving as an energy source for hematopoietic stem cells. These findings suggest that the functions of human skull bone marrow and SMCs may be more complex and significant compared to those of mice [39, 41–43].

With the discovery of SMCs, the traditional cognition that the immune cells of the CNS mainly come from the peripheral hematopoietic system, such as the tibia, has been gradually broken. Increasing evidence demonstrates that SMCs can transport immune cells from the skull bone marrow to brain tissue. Simultaneously, CSF can deliver “danger signals” from brain tissue to skull bone marrow, creating a vicious cycle.

## Distinction between skull bone marrow and other bone marrow to CNS

CNS-resident immune cells and peripheral circulation immune cells are separated by the BBB [44], and the crosstalk between the CNS and peripheral immune systems is limited under steady-state conditions but increases during pathologies [45–47]. The bone transcriptome can be changed in pathological conditions. Different regions of the bone marrow exhibit unique characteristics, with skull bone marrow showing the most pronounced molecular heterogeneity, including distinct transcriptomic and proteomic signatures in neurological pathologies [39]. Recent studies have found that meninges-derived immune cells are distinct from blood-derived immune cells of the same type and seem to have more surprising functions [30].

The skull bone marrow plays a critical role in CNS immunity by preferentially receiving stimulatory signals transmitted through CSF. On the one hand, CSF provides a protective microenvironment by transporting metabolic waste from the brain to the peripheral circulation system through meningeal lymphatic vessels [20, 21], as well as arachnoid villi [48], ultimately causing a systemic adaptive immune response [49, 50]. It is suggested that CSF may be an important messenger that regulates neuro-immunity. Moreover, researchers have confirmed that peripheral bone marrow-derived macrophages can accelerate the recovery of spinal cord injury and stroke [47, 51]. However, the skull, the bone closest to the CNS, also contributes to the CNS immune response. With the discovery of SMCs, Fadi E et al. found that CSF can bidirectionally communicate with skull bone marrow through SMCs in mice with bacterial meningitis [33].

Comparative transcriptomic profiling demonstrates distinct immunological signatures between skull and peripheral bone marrow compartments, revealing significant heterogeneity in their molecular characteristics. Skull bone marrow-derived immune cells show reduced expression of pro-inflammatory mediators (including IL-6 and TNF-α) and chemokines (such as CCL5 and CXCL9), while upregulating genes related to metabolic regulation, inflammation suppression, and tissue repair processes, demonstrating primary immunomodulatory and regenerative functions; in contrast, peripheral bone marrow immune cells enhance the expression of genes related to leukocyte migration, T cell activation, and pro-inflammatory cascade reactions, showing stronger inflammatory capabilities [30]. Single-cell RNA sequencing (scRNA-seq) analysis further confirms that among three groups of mice (naïve, MCAo, and sham surgery), skull bone marrow shows the most differentially expressed genes compared to bone marrow from other sites, with these changes primarily manifesting in neutrophils (for detailed information, see the “STROKE” section [39, 52]).

Proteomic analyses demonstrate that skull bone marrow exhibits distinct characteristics, particularly regarding neutrophil functionality. Animal studies revealed differential protein expression patterns in the skull, predominantly associated with extracellular matrix organization, neutrophil degranulation, immune response mechanisms, and protein transport pathways, Corroborating these findings, human studies identified that skull bone marrow harbors a unique proteome profile, with proteins primarily involved in chemical synaptic transmission and immune cell regulation, showing concordance with the murine model results [39, 53]. Additionally, COL1A1 is the most abundant protein in the human skull, covering the blood vessels in the bone marrow. This indicates that the vessels in the skull bone marrow are different from those in other bones. This indicates that the vessels in the skull bone marrow are different from those in other bones (for detailed information, see the “STROKE” section [54]).

In summary, these findings underscore the unique role of skull bone marrow in CNS immunity and its immunomodulatory potential in neuropathological conditions. Future studies should investigate the bidirectional communication mechanisms between skull bone marrow and CSF, as well as their specific regulatory effects on neuro-immunity. Such research could inform the development of targeted therapeutic strategies, including modulation of CSF signaling or skull bone marrow immune cells, to enhance treatment outcomes for neurological disorders.

## The main methods for verifying different bone marrow-derived cells

### Bone marrow tagging using the microinjection technique

**Fig. 3:**
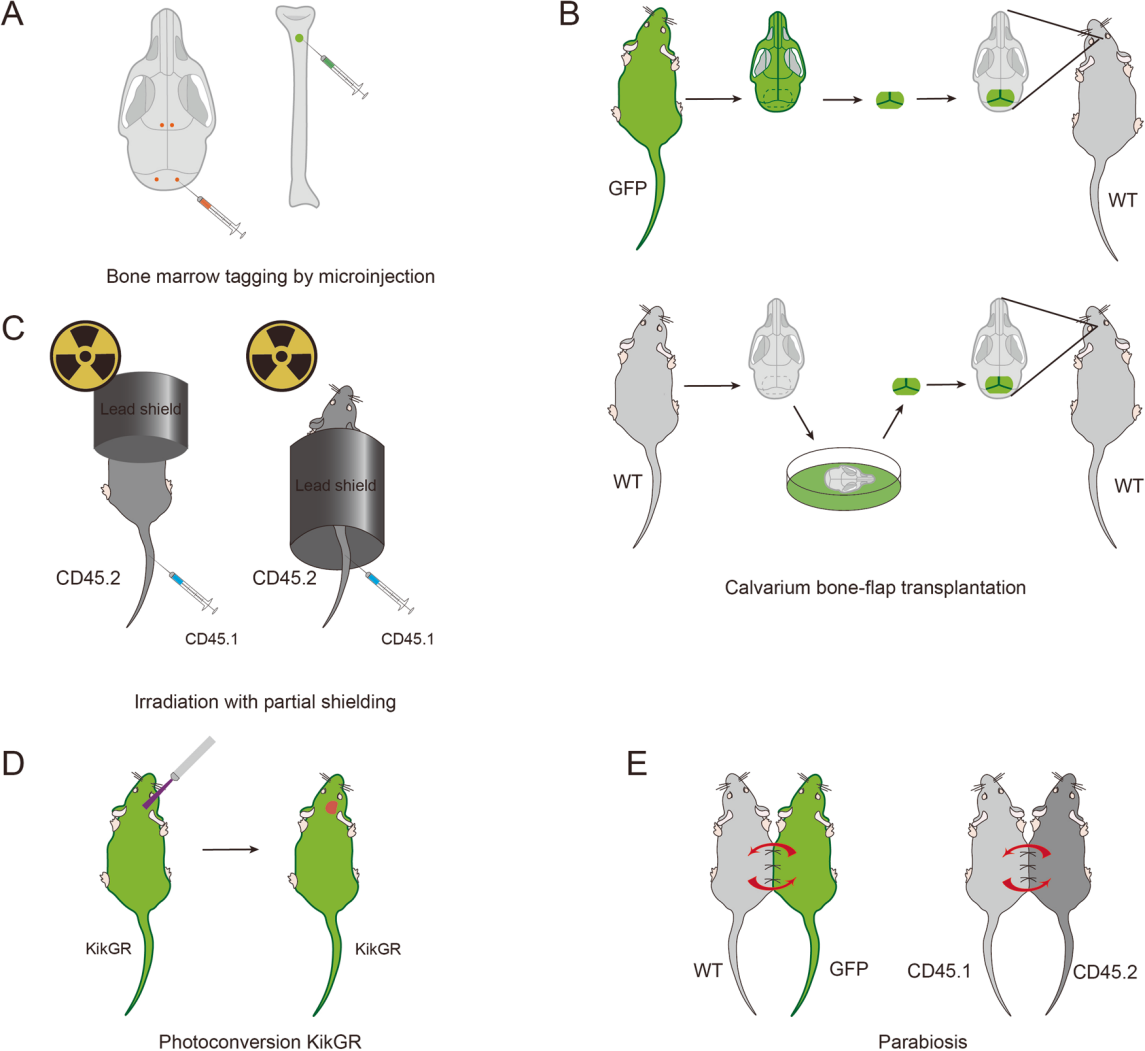
Primary methods for studying immune cells derived from various bone marrow sources. **A** Using microinjection technology to inject dyes of different colors into the bone marrow of the skull and tibia, respectively. **B** Transplantation of colored skull bone marrow into the bone windows of wild-type or differently colored mouse skulls. **C** Radiation technology to ablate the hematopoietic function of CD45.2^+^ mouse skull bone marrow, followed by the injection of CD45.1^+^ bone marrow cells into the tail of the mouse, and subsequent verification of CD45.1^+^ cell content in the central nervous system of CD45.2^+^ mice. **D** Using KikGR gene mice, where UV laser irradiation converts green fluorescent protein in the mouse skull to red fluorescent protein. **E** Parabiosis surgery on two mice, each labeled with different methods for immune cells (left: GFP-WT, right: CD45.1+−CD45.2+), to establish peripheral circulation.

The initial discovery of SMCs and the confirmation that the main source of myeloid cells in the brain parenchyma is skull bone marrow were accomplished using bone marrow microinjection labeling [25]. Researchers microinjected deep red and green dyes into the skull bone marrow and tibia, respectively, under sterile conditions. Four sites were selected at bilateral frontal and occipital locations, and the tibial injection site was at the muscle insertion below the knee. Flow cytometry was used to analyze the red-labeled and green-labeled cells in brain tissue after the designated period [25] (Fig. 3A).

### Skull bone-flap transplantation

Researchers used an electric drill to create a 4 mm by 6 mm bone window on the parietal and interparietal bones of wild-type mice. After separating from the surrounding bones, the skull was removed using micro forceps without damaging the underlying meninges, and the brain was then rinsed with a large amount of 0.9% sodium chloride. Additionally, fluorescent mice of the same sex were selected, and skull bones of the same size were harvested. After peeling off their dura mater, the skull bones were placed on the bone window of wild-type mice, then sutured, and injected with painkillers and antibiotics daily until the 5th day [30]. Wild-type mice can be replaced with fluorescent mice of different colors from donor mice, allowing for simultaneous observation of the number of immune cells derived from both systemic circulation and skull bone marrow [55] (Fig. 3B).

### Irradiation with partial shielding

Experimental animals (e.g., mice) are exposed to a lethal dose of total body irradiation (typically 9–12 Gy) to ablate the host bone marrow and peripheral hematopoietic and immune cells, thereby suppressing the host immune system. Simultaneously, specific anatomical regions (e.g., the head, limbs, or skin) are protected from radiation damage using shielding devices made of high-density materials, such as lead plates, to preserve the resident immune cells or hematopoietic stem cells in those areas. Within 24 h after irradiation, donor-derived bone marrow cells (typically 5 × 10^6^ to 1 × 10^7^ cells) are transplanted via tail vein injection to reconstitute the host immune system. The distinction between donor and host cells can be achieved using genetic markers (e.g., the CD45.1/CD45.2 congenic system) or fluorescent labeling (e.g., GFP-labeled donor cells). At the end of the experiment, tissues from both shielded and non-shielded regions are collected for analysis. Techniques such as flow cytometry, immunofluorescence staining, and molecular biology assays (e.g., qPCR or RNA sequencing) are employed to evaluate the distribution, proliferation, and functional characteristics of donor-derived cells and resident cells [30, 56] (Fig. 3C).

### Photoconversion KikGR

Kikume Green-Red (KikGR) mice are a transgenic mouse model that expresses green fluorescent protein throughout their bodies. After establishing a disease model, such as stroke [39], the skull is exposed under sterile conditions and irradiated with a 5 mm diameter UV light beam positioned 5 cm above the affected side of the skull for 3 min. This induces photoconversion, converting the green fluorescent protein into red fluorescent protein. The mouse scalp is then sutured, and the experiment proceeds for the required duration [39, 57]. Using this method, researchers can detect red fluorescent-labeled immune cells, including B cells, T cells, and myeloid cells, in the affected side of the brain. This approach enables the tracking of immune cell migration pathways, such as the migration of myeloid cells after brain injury, confirming their origin in the skull bone marrow. Thus, the KikGR mouse model provides a powerful tool for studying immune cell communication between the skull bone marrow and the brain (Fig. 3D).

### Parabiosis

Shave and disinfect the corresponding surfaces of two mice. Make a skin incision from the olecranon to the knee joint, bluntly separate the subcutaneous fascia, and form a 0.5 cm free skin flap. First, use a 5-0 Vicryl suture to sew the olecranon and knee together to reduce skin tension. After pressing, use a 5-0 nylon suture to sew the heterozygous partner’s dermis and skin incision [30]. And also injected with painkillers and antibiotics daily. According to experimental requirements, choose either one wild-type mouse and one fluorescent mouse, or two fluorescent mice of different colors (Fig. 3E).

Research methods for studying bone marrow-derived cells have unique characteristics tailored to specific research objectives, but they also have limitations that require integration with advanced technologies to achieve comprehensive biological insights. Bone marrow tagging using the microinjection technique enables dynamic tracking of bone marrow-derived cell migration by injecting fluorescent markers into the bone marrow cavity. It is relatively simple to perform but has limited applications due to low labeling efficiency and the inability to resolve molecular characteristics. Skull bone-flap transplantation facilitates long-term studies of locally resident cells by transplanting labeled tissue to distinguish between host and donor cells. This method is suitable for exploring cellular functions in specific tissue microenvironments but involves complex surgical procedures and risks of immune rejection. Irradiation with partial shielding uses radiation with local shielding to simulate natural hematopoietic reconstruction processes, allowing for comparisons of functional differences between cells in different regions. However, it requires complex experimental designs and carries the risk of radiation side effects. Photoconversion KIKGR provides high spatiotemporal resolution for cell labeling and tracking via photoconvertible proteins. It is ideal for studying cell dynamics at specific time points and spatial locations but relies on transgenic models and specialized equipment, limiting its accessibility. Parabiosis investigates bone marrow-derived cell migration, homing, and function through shared blood circulation, distinguishing between locally resident and circulating cells while preserving the natural microenvironment. It is suitable for long-term observation and simulating physiological conditions but involves complex operations, is prone to blood factor interference, has limited molecular resolution, and requires strict matching of experimental animal characteristics to avoid immune rejection.

In contrast, advanced technologies such as scRNA-seq and high-resolution imaging provide significant advantages in molecular characterization and spatial distribution studies. scRNA-seq reveals gene expression, epigenetic states, and functional statuses of cells, while high-resolution imaging maps cellular spatial positions and interactions within tissues with precision. However, these technologies primarily offer static information and cannot capture dynamic cell migration or functional changes.

To overcome these limitations, combining traditional methods with advanced technologies can provide a more comprehensive understanding of bone marrow-derived cells. This integration enables dynamic behavior analysis, molecular mechanism exploration, and spatial distribution studies, advancing research across multiple dimensions and deepening our understanding of these cells.

## Skull bone marrow in neurological disease

Immune cell recruitment is the basis of secondary injury to the CNS [58–60]. Anatomical differences in the CNS determine the composition of immune cells in homeostasis and under different pathological conditions [61–63]. The principle of proximity contributes to the rapid responsiveness and specificity of skull bone marrow in response to CNS injury and challenges the idea of uniform leukocyte release into the systemic circulation [25]. Moreover, the skull bone marrow can produce varying hematopoietic responses depending on the stimulation signals (Table 2).

**Table 2:** The impact of various immune cell types on CNS diseases.

Disease	Cell type	Characteristic
MS	Myeloid cells	Monocyte entry into the brain and spinal cord is required for progression to severe stages of EAE [156].
		The neutrophil-to-lymphocyte ratio is positively correlated with patient prognosis [157].
	Lymphocytes	Autoreactive T cells enter the bone marrow through the CXCL12–CXCR4 axis, promoting bone marrow regeneration and disease occurrence, they also promote increased neutrophil and ly6c^high^ monocyte production through the CCL5–CCR5 axis, promoting disease progression [156].
		B cells are significantly enriched in the CSF and brain tissue of MS patients [158].
		Natural killer (NK) cells inhibit T cell proliferation, kill autoreactive T cells, suppress the differentiation of myelin-reactive T helper type 1 (Th1) and type 17 (Th17) cells in the CNS, and secrete neurotrophic factors [159–164].
	Lymphocytes	The number of CD8^+^ T cells significantly increases in cerebral ischemic tissue [165–169].
AD	Myeloid cells	The ability of monocytes to engulf amyloid beta (Aβ) is impaired, and they can release cytotoxic factors that disrupt the BBB [170]. Neutrophils promote AD-like pathology and cognitive decline through LFA-1 integrin [171].
	Lymphocytes	B cells alleviate Aβ load and cognitive impairment in 5×FAD mice by producing IL-35 [172]. Increased activated CD8^+^ T cells are found in the CSF and blood of mild AD patients [173].
.PD	Myeloid cells	Impaired phagocytic ability of monocytes and macrophages towards α-synuclein [174, 175].
	Lymphocytes	CD4^+^CD8^+^ T cells can respond to α-synuclein to produce Th1/Th2 cytokines, but the impact of CD4^+^ T cells that produce IFN-γ is greater than that of CD8^+^ T cells. CD4^+^ T cells can promote neuronal degeneration [176].
		B cells play a dual role in PD, functioning both to inhibit inflammation by releasing anti-inflammatory factors via regulatory B cells and to promote neuroinflammation by activating T cells and releasing antibodies through pro-inflammatory B cells [38, 118].
		In preclinical mouse models of PD, NK cells have been shown to be scavengers of α-synuclein. Systemic depletion of NK cells exacerbates α-synuclein synaptic nuclear protein pathology, while systematic clearance of α-synuclein can alleviate the pathological changes caused by α-synuclein [177]
Aging	Myeloid cells	Aging is accompanied by an increase in neutrophils. Due to enhanced PI3K signaling within cells, chemotaxis is weakened, and phagocytic bactericidal ability is impaired [178–181].
		During aging in mice and humans, the production of classical monocytes expressing MHCII increases [182].
	Lymphocytes	Decreased NK cell numbers in multiple organs of elderly mice [183].
ALS	Myeloid cells	The neutrophil-to-lymphocyte ratio is negatively correlated with the survival time of ALS patients [184].
	Lymphocytes	A high frequency of CD4^+^FOXP3- effector T cells in blood and CSF is associated with a low survival rate, while a high frequency of activated regulatory T (Treg) cells in blood and a high ratio between activation and resting states are associated with a better survival rate [185].
Brain injury	Myeloid cells	Sudden adrenergic activation following acute brain injury biases bone marrow HSCs toward the myeloid lineage, resulting in increased production of Ly6C^low^ patrol monocytes. These monocytes migrate into the injured brain and reduce neuroinflammation [52].
		Skull bone marrow-derived neutrophils rapidly migrate to brain tissue, releasing NETs and inflammatory factors, exacerbating neuroinflammation [3, 25].
Major depressive disorder	Myeloid cells	Under long-term chronic stress, the sympathetic nervous system in the bone marrow releases norepinephrine, inhibiting CXCL12 expression and thereby promoting the release of monocytes and neutrophils [186–191].
		Ly6C^high^ monocytes and neutrophils release MMP8, which controls the ultrastructure of the extracellular space of neurons, thereby affecting neuronal function [192].
	Lymphocytes	Reduction in CD4^+^CB^+^ T cells is associated with an enhanced immune inflammatory response system in major depressive disorder [193].

### Multiple sclerosis

Multiple sclerosis (MS) is an autoimmune disease characterized by CNS demyelination and significant immune cell infiltration [64]. Among the infiltrating immune cells, T cells are the dominant type [65–71]. Autoreactive T cells recruit hematogenous myeloid cells, primarily monocytes and neutrophils, by inducing a cascade of cytokines and chemokines. These monocytes and neutrophils further aggravate neuroinflammation by releasing cytokines, chemokines, and reactive autoreactive T cells, eventually forming a vicious cycle [65, 70, 72, 73].

The maintenance of hematopoietic function in bone marrow is fundamental for sustaining inflammatory responses. [25, 28, 30, 74] In the experimental autoimmune encephalomyelitis (EAE) mouse model, T cells mediate a substantial proliferation of diverse cellular populations within the bone marrow, including hematopoietic stem cells and Ly6C^high^ monocytes, through CCL5–CCR5 signaling pathways. Notably, CXCL10^+^ monocytes demonstrate pathogenic properties in this context. While traditional multiple sclerosis research has predominantly focused on T cell-mediated mechanisms, emerging evidence, particularly regarding SMCs and skull bone marrow hematopoiesis, has revealed a more comprehensive understanding of the cellular sources contributing to central nervous system infiltration [75].

Cugurra et al. systematically analyzed the functional heterogeneity and molecular mechanisms of myeloid cells derived from skull and vertebral bone marrow versus peripheral blood in CNS inflammation using the EAE model. Their findings revealed distinct functional and dynamic patterns of myeloid cells in CNS inflammation [30]. Myeloid cells from skull and vertebral bone marrow migrate to CNS boundary regions (e.g., dura mater) and parenchyma through SMCs, exhibiting an immunosuppressive phenotype. scRNA-seq showed that skull marrow-derived myeloid cells express low levels of pro-inflammatory cytokine genes (IL-6, IL-1β, TNF-α) and chemokine genes (CCL2, CCL5, CXCL9, CXCL16) while upregulating genes associated with metabolic regulation, lipid transport, inflammation suppression, inflammatory residue clearance, and tissue repair. In contrast, peripheral blood-derived myeloid cells exhibit a strong pro-inflammatory phenotype upon entering the CNS through blood circulation. Transcriptome analysis revealed high expression of genes related to leukocyte migration, cell adhesion molecules, and T cell activation, making these cells key drivers of inflammatory cascades and tissue damage. Spatial distribution analysis confirmed that skull bone marrow-derived myeloid cells primarily localize to CNS boundary regions, where they limit inflammation spread through their immunosuppressive functions during acute EAE. Conversely, peripheral blood-derived myeloid cells preferentially infiltrate CNS parenchyma, amplifying inflammation and causing tissue damage. Spatial distribution analysis further confirmed that skull bone marrow-derived myeloid cells mainly distribute in CNS boundary regions, limiting inflammation spread through their immunosuppressive function during acute EAE; while peripheral blood-derived myeloid cells tend to infiltrate CNS parenchyma, mediating inflammation amplification and tissue damage. Temporal dynamic studies showed these two cell types undergo functional conversion during disease progression: the acute phase is dominated by peripheral blood-derived myeloid cell-mediated inflammatory response, while the chronic phase is characterized by skull bone marrow-derived myeloid cell-mediated inflammation clearance and tissue repair [30]. Consistently, TSPO-PET imaging in patients with primary progressive MS and relapsing-remitting MS showed significant signal enhancements in the temporal pole of the skull [38].

The role of skull bone marrow and its myeloid cells in MS extends beyond traditional understanding. Their dynamic regulatory functions during both acute and chronic phases, direct connection to the central nervous system CNS, and immunosuppressive properties redefine the immune regulatory network involved in CNS inflammation. This discovery not only enhances our understanding of the pathological mechanisms underlying MS but also paves the way for novel immune intervention strategies in the context of precision medicine. Future research should focus on elucidating the cross-tissue signaling mechanisms between skull bone marrow and the CNS, as well as developing targeted approaches to regulate skull bone marrow-derived myeloid cells for comprehensive intervention and management of MS progression.

### Stroke

Stroke is a focal neurological deficit resulting from cerebral vascular obstruction, rupture, or spasm, leading to impaired blood flow and subsequent brain tissue damage [76]. Emerging evidence suggests that skull bone marrow plays a distinct and crucial role in stroke [25, 30]. Additionally, a previous study confirmed that skull bone marrow-derived neutrophils and monocytes are present in the infarct lesion within 6 h after injury using cell tracking [25]. To reduce the effects of dye leakage resulting from bone marrow tagging via the microinjection technique, Zeynep et al. converted the cells in the skull bone marrow to RFP^+^ cells using ultraviolet laser illumination in a KikGR mouse model. Equivalent results were obtained, with RFP^+^ cells observed in the ipsilateral brain parenchyma at 1 and 6 h after stroke. This further supports the preferential nature of immune cells released from the skull bone marrow. Hierarchical clustering showed that the skull bone has its own branch in the MCAo condition compared to sham and naïve conditions, implying the unique strategic position of skull bone marrow in stroke [39].

The skull bone marrow undergoes significant cellular and transcriptomic changes following stroke, highlighting its unique role in the immune response. Using the vDISCO tissue-clearing technique, cellular-resolution imaging of the mouse skull revealed an increase in total skull bone marrow cell numbers post-stroke [77]. However, real-time imaging indicated a reduction in LysM^+^ cells, suggesting that specific cell types, particularly myeloid cells, are mobilized from the skull, accompanied by associated transcriptomic shifts [39]. Notably, the level of SDF-1, a leukocyte retention factor that restricts neutrophil release from hematopoietic niches, significantly decreases in the skull bone marrow within six hours of stroke [25, 78]. partially explaining this stark contrast. scRNA-seq analysis of bone, dura mater, and brain cells from three groups of mice (naïve, MCAo, and sham surgery) demonstrated that the skull bone marrow exhibited the highest number of differentially expressed genes compared to other bone marrow. Specifically, 96, 15, and 62 genes were upregulated, while 138, 538, and 62 genes were downregulated in the three respective groups, with neutrophils being the primary cell type affected. Analysis of neutrophil developmental trajectories revealed that, under naïve conditions, meningeal neutrophils closely resembled those from various bone marrows. However, under sham surgery and MCAo conditions, meningeal neutrophils were most similar to those derived specifically from the skull. Following MCAo, shared differentially expressed genes between the dura mater and skull increased from 6 upregulated and 7 downregulated genes to 29 upregulated and 15 downregulated genes [39]. These findings suggest that immune cells, particularly neutrophils, migrate from the skull bone marrow to the brain during neuroinflammatory events, as evidenced by the comparison of meningeal and skull bone marrow cell characteristics [39, 52].

Proteomic analyses further underscore the ©unique features of skull bone marrow, particularly in neutrophil-related functions. Compared to other bone marrow, the mouse skull exhibited increased expression of 45, 65, and 67 proteins under three different conditions, with a greater number of proteins showing decreased expression [39, 53]. Functional classification of these proteins identified three main categories: (1) extracellular matrix (ECM) composition, (2) neutrophil degranulation and immune response, and mRNA processing, and (3) protein transport, neutrophil degranulation, and immune pathways [39, 53]. These findings underscore that neutrophil-related functions distinguish the skull from other bone marrow niches. Similarly, proteomic analysis of human bone specimens showed that the skull bone marrow expressed the highest number of proteins, including 105 unique to the skull. These proteins were primarily associated with chemical synaptic transmission and ECM functions involved in immune cell activity. Interestingly, human skull protein functions, such as neutrophil degranulation and mRNA processing, align closely with those observed in mice [39, 53].

Recent research highlights the unique and indispensable role of skull bone marrow in the immune response following stroke, particularly in neutrophil mobilization, migration, and functional regulation. Unlike other bone marrow sources, skull bone marrow exhibits distinct cellular, transcriptomic, and proteomic characteristics. Its anatomical proximity to the meninges positions it as a critical hub for post-stroke immune responses. This distinctiveness underscores the strategic importance of skull bone marrow in neuroinflammation and provides valuable insights into the dynamic interactions within the brain–bone marrow immune axis after stroke. Future studies should investigate the molecular mechanisms underlying skull bone marrow’s role in post-stroke immune regulation and assess its potential for therapeutic applications, such as targeting skull bone marrow-derived immune cells to improve stroke outcomes. Advancing research in this area could significantly enhance our understanding of stroke pathology and contribute to the development of precision medicine, offering new therapeutic opportunities for patients.

### Traumatic brain injury

Immune cell recruitment plays a crucial role in secondary CNS injury [58–60, 79]. As the first line of host immunity, neutrophils are the first cell type to reach the injury lesion [3, 9]. The infiltration of neutrophils is a hallmark of sterile neuroinflammation. Additionally, neutrophil extracellular traps (NETs) have been closely associated with secondary immunothrombosis following CNS injury [5, 80–84]. Using a mouse chimera model, Cugurra, A. et al. found that the proportion of Ly6C^+^ monocytes and neutrophils infiltrating the optic nerve was reduced compared to the peripheral circulation during the same period. In contrast, analysis of infiltrating cells at the puncture site of the ear skin, a peripheral injury model, was consistent with the extent of chimerism in peripheral blood [30]. This indicates that the source of CNS-infiltrating cells is primarily the skull bone marrow, rather than peripheral bone marrow. This finding has been corroborated in a model of spinal cord injury, as detailed in the section on MS.

Traumatic brain injury involves primary brain damage from external forces and secondary injury driven by inflammatory responses, resulting in short- or long-term behavioral and cognitive impairments [85]. A recent study on TBI found that the sources of immune cells infiltrating brain injury foci are divided into skull bone marrow and peripheral bone marrow. The two types of immune cells from different sources differ in quantity, distribution, and function, distinguishing them from other neurological diseases without direct disruption of the BBB [86]. Researchers indicated that the infiltrating cells are primarily immune cells, particularly Ly6G^+^ neutrophils and CCR^+^ monocytes. In terms of quantity, peripheral bone marrow-derived cells are more than twice as numerous as skull bone marrow-derived cells. In terms of distribution, skull bone marrow-derived cells are mainly located in the edge area of the trauma lesion, while peripheral bone marrow-derived cells are mainly located in the core area of the trauma lesion. In terms of function, skull bone marrow-derived cells mainly express the anti-inflammatory cell phenotype of Arg^+^, while peripheral bone marrow-derived cells mainly express the pro-inflammatory cell phenotype of CD86^+^. Moreover, skull bone marrow-derived cells exhibit stronger efferocytosis ability, expressing the “eat me” signal receptor MerTK to phagocytose cell debris and reduce inflammatory reactions [55]. The results of this study are groundbreaking, which uniquely demonstrates that skull bone marrow cells infiltrate the brain following TBI and may influence its pathogenesis. These cells appear to play distinct roles compared to peripherally derived immune cells within the neuroinflammatory environment, offering potential pathways for targeted therapeutic interventions. However, the researchers used a controlled cortical impact (CCI) instrument to create the TBI model, which directly damages the skull bone marrow cavity and SMCs. This undoubtedly affects the number and transport of cells in the bone marrow of the skull to a certain extent, and even to a large extent. Although CCI is currently the main method for creating TBI models, it is unknown whether using a closed injury model would have a disruptive effect on the proportion of cells derived from skull bone marrow and peripheral bone marrow.

### Subarachnoid hemorrhage

Subarachnoid hemorrhage (SAH) is a type of intracranial hemorrhage caused by the rupture of intracranial blood vessels, leading to blood flow into the subarachnoid space, most commonly due to intracranial aneurysm rupture [87, 88]. The immune-inflammatory response is a key pathological mechanism underlying secondary brain injury after SAH [89–91]. As a critical immune cell reservoir for the CNS, skull bone marrow plays a pivotal role in the immune response following SAH.

Research has shown that skull bone marrow is a major source of Ly6C^high^ monocytes, contributing significantly more than limb bone marrow [92]. After SAH, microglia secrete elevated levels of the chemokine CCL2, generating strong chemotactic signals that drive Ly6C^high^ monocytes from skull bone marrow to migrate across the BBB into brain tissue. Due to its anatomical proximity to the CNS, skull bone marrow can rapidly respond to brain inflammatory signals, providing immune cells more efficiently than limb bone marrow [92].

These Ly6C^high^ monocytes exhibit dual roles in the brain. During the acute phase (1–3 days post-SAH), they differentiate into monocyte-derived dendritic cells (moDCs), releasing pro-inflammatory cytokines such as TNF-α to enhance immune responses and clear damaged tissue. In the subacute phase (5–7 days post-SAH), they further differentiate into macrophages with anti-inflammatory and tissue-repair functions, promoting neurological recovery. This unique functional capacity underscores the role of skull bone marrow as a critical immune cell supplier for the CNS. Experimental studies using bone marrow chimera models or CCL2 signaling blockade demonstrated that depletion of bone marrow cells or inhibition of CCL2 significantly reduced Ly6C^high^ monocyte infiltration into the brain, impairing neurological recovery. These findings further confirm the central role of skull bone marrow in Ly6C^high^ monocyte migration and brain injury repair after SAH [92].

Conversely, another study found that neutrophils derived from skull bone marrow skull bone marrow-derived neutrophils in aSAH mice have a stronger ability to produce NETs than peripheral-derived neutrophils, and the threshold potential to induce NETosis in vitro is lower [93]. This indicates that skull bone marrow-derived neutrophils mainly play a harmful role in SAH mouse models.

The immune response of skull bone marrow following SAH exhibits both protective and potentially harmful effects. On the one hand, skull bone marrow-derived Ly6C^high^ monocytes contribute to clearing damaged tissue and promoting neurological recovery. On the other hand, neutrophils derived from skull bone marrow may aggravate inflammation and brain injury through excessive formation of NETs. These findings highlight the need for future research to further investigate the specific functions and regulatory mechanisms of distinct immune cell subsets in skull bone marrow. Such studies could pave the way for more precise therapeutic strategies, such as optimizing SAH treatment outcomes by modulating CCL2 signaling or inhibiting NET formation.

### Central nervous infection

Distinct from sterile neuroinflammation, the occurrence of meningitis is mainly related to pathogen invasion, including bacteria and viruses. Non-resident tissue can survive longer in CNS compared to peripheral organs, called CNS immune privilege. While the viewpoint is being overturned due to the discovery of CNS lymphatic vessels, implying that the CNS is monitored continuously by systemic immunity through the CSF-dCLNs communication [21]. Under normal conditions, CSF flows along the perivascular spaces of the dura mater and enters the bone marrow cavity through SMCs. CSF flow tracers are detected in the perivascular spaces of the bone marrow. High-resolution microscopic imaging, tissue clearing, and fluorescent tracing techniques confirmed that CSF is primarily concentrated in the skull bone marrow region and does not diffuse to distal bone marrow, such as the tibia. In a bacterial meningitis model, streptococci enter the skull bone marrow via CSF, predominantly residing near the SMCs and spreading further into the bone marrow through perivascular spaces. Bacterial infection significantly induces the proliferation of HSCs and myeloid progenitor cells in the cranial bone marrow, while also promoting the migration of myeloid cells to the meninges to participate in immune defense. Furthermore, the study found that bacteria in CSF activate bone marrow hematopoiesis through the Toll-like receptor (TLR) signaling pathway, with this response occurring earlier and more robustly in skull bone marrow than in distal bone marrow. Gene knockout experiments using Myd88-deficient mice showed that the absence of TLR signaling significantly weakens this immune response, indicating that CSF plays a critical role as a signaling pathway in infection and inflammation. This study is the first to reveal that CSF is not only a medium for waste clearance and signal transmission but also regulates local immunity and hematopoietic function during CNS inflammation and infection [33].

Skull bone marrow, as a hematopoietic tissue capable of producing immune cells, can preferentially supply leukocytes to the meninges to support local immunity before systemic immunity during meningitis. Thus, targeting the skull bone marrow as a new organ for treating meningitis while reducing the use of systemic antibiotics can maximize control of infection progression and reverse neurological deficits.

## Neurodegeneration diseases

The immune system plays a crucial role in the progression of neurodegenerative diseases [94]. The CNS is surrounded and protected by the dense and tough outer layer of the skull, traditionally believed to be solely a physical protective structure. However, an interesting study found that the shape of the skull is correlated with neurodegenerative diseases [95]. Consistent with this, an anatomical study indicated irregular thickening of the inner surface of the frontal bone in patients with Parkinson’s disease (PD), Alzheimer’s disease (AD), and depression [96]. However, in the relevant research on skull shape, researchers only found correlations and did not further investigate causal relationships.

AD and PD are two of the most common neurodegenerative disorders [97] Amyloid beta (Aβ), phosphorylated tau (p-tau), and total tau (T-tau) are closely related to the development of AD. They lead to neuronal tangles, which in turn cause neuronal synaptic loss, neuronal morphological changes, and even neuronal death [97–100]. And Aβ, p-tau, and T-tau have also become CSF biomarkers for the diagnosis of AD [101]. Additionally, many new potential biomarkers are being established, including SNAP25, synaptotagmins, neuronal calcium-sensing protein VLP1, the promising neurogranin, and some non-protein biomarkers [102–106]. Following the discovery of meningeal lymphatic vessels, the pathogenicity of these toxins in CSF for AD has been further confirmed [107]. Previous studies on AD have primarily focused on peripheral immunity. Research on immune cells in the blood indicates that peripheral immune cells contribute to neuroinflammation and disease progression through various mechanisms. Neutrophils exacerbate neuroinflammation and impair cerebral blood flow by releasing NETs, reactive oxygen species (ROS), and inflammatory factors.

CD4^+^ T cells release inflammatory mediators that activate microglia, while CD8^+^ T cells induce neuronal damage through cytotoxic effects. B lymphocytes secrete antibodies to clear amyloid-beta (Aβ), but they may also activate the complement system, thereby aggravating inflammation. Natural killer (NK) cells amplify inflammatory responses through direct cytotoxicity and the release of inflammatory factors. Monocytes, while protective in the early stages by clearing Aβ and regulating inflammation, exhibit reduced functionality in later stages due to metabolic disturbances, further exacerbating the disease. These complex cellular mechanisms represent potential therapeutic targets for AD treatment [108]. In addition, increasing evidence highlights the critical role of skull bone marrow in CNS immunity, as it provides immune cells to the CNS in a rapid and targeted manner. Notably, regions of elevated TSPO-PET signals in the skulls of AD patients (covering the temporal and parietal cortical frontal skull) align with affected brain regions (motor and frontoparietal cortex). Longitudinal TSPO-PET monitoring of AD patients has revealed that late-stage patients exhibit higher TSPO-PET signals compared to early stages, indicating that skull inflammation persists throughout all stages of AD [39]. Furthermore, the established bidirectional communication between CSF and skull bone marrow [28, 33], which suggests that toxins in the CSF may infiltrate the skull bone marrow niche via SMCs, promoting hematopoiesis and influencing CNS inflammation. Furthermore, while heterologous experiments have shown that circulating monocytes cannot be implanted into the CNS of AD mice, and scRNA-seq has revealed the presence of monocytes in the brain, further confirming that skull bone marrow can transport immune cells to the brain of AD mice through non-circulating blood pathways [109–111].

The role of skull bone marrow in AD has begun to emerge, but research in this area remains in its early stages. Future studies should focus on gathering more experimental and clinical data to elucidate its specific mechanisms and therapeutic potential. Key areas of investigation include examining functional changes in skull bone marrow immune cells at different stages of AD, understanding how CSF toxic factors influence skull bone marrow through SMCs, and developing therapeutic strategies targeting skull bone marrow immune regulation. In conclusion, the immune interactions between skull bone marrow and AD offer a novel perspective for understanding the complex pathological mechanisms of the disease and provide promising directions for the development of innovative therapeutic approaches.

PD is a neuroinflammatory disease caused by the abnormal accumulation of α-synuclein in the brain [112]. This accumulation can lead to different diseases or subsets, such as multiple system atrophy (MSA), mainly due to the distinct conformers of misfolded α-synuclein [113, 114]. Studies have shown that α-synuclein, as an activator of lymphocytes, can disrupt the balance of pro-inflammatory and regulatory lymphocyte subsets in PD patients and experimental models [115–120]. Consequently, research on antibodies targeting α-synuclein has emerged. Researchers found that total α-synuclein antibodies were elevated in prodromal PD patients, who had a higher risk of developing PD [118, 119, 121]. This suggests that α-synuclein antibodies may be linked to the progression of CNS inflammatory diseases, including PD. However, the complexes formed by these antibodies and α-synuclein can be recognized and internalized by microglia for degradation, promoting the clearance of pathological α-synuclein [122]. The mechanisms involved are complex, as high-affinity and low-affinity antibodies have different clearance effects on α-synuclein [121, 123]. Therefore, further research is needed on antibody formation and its role.

As precursor cells of antibody-producing plasma cells, B cells have various subgroups and markers. For instance, regulatory B cell subsets can inhibit T cells and innate immune activation by releasing cytokines like IL-10 and IL-35, playing a protective role in PD development. In contrast, memory B cell subsets can promote inflammatory responses by activating T cells through antigen presentation [118, 124]. In PD patients, peripheral total B lymphocytes are decreased, with a more significant reduction in regulatory B lymphocytes, while the proportion of pro-inflammatory B lymphocytes is increased [120, 125]. This indicates that B lymphocytes play a complex role in PD, participating in immune regulation and disease progression through multiple pathways [124]. Recent research has found that in healthy conditions, most B cells in the dura mater originate from the skull bone marrow [38, 118]. Unlike blood-derived B cells, which are all mature, the dura mater contains B cells at various developmental stages, from pre-B cells to mature B cells [119]. The microenvironment differs from the periphery, and the meninges provide a specific environment for B cells, eliminating self-reactive B cells against self-antigens through negative selection and offering additional protection against CNS diseases [126]. Under pathological conditions, these B cells can form meningeal ectopic lymphoid structures, responding rapidly and accurately to CNS antigens and signals, and playing a role in local immune surveillance [127].

This prompts us to explore whether there is a correlation or consistency between B cell aggregation areas in the meninges and high-signal regions in the skull indicated by TSPO-PET.

In summary, B cells exhibit complex and diverse roles in PD, with their regulatory mechanisms in both peripheral and central immune systems offering valuable opportunities for advancing disease research and treatment. Future studies should aim to further elucidate the functions and dynamic changes of B cell subsets while integrating imaging techniques with immunological analyses to uncover the specific roles of B cells in disease onset and progression. Such efforts will contribute to the development of precision medicine approaches for PD. Additionally, regarding cellular function, cells from different sources, including B cells, cannot be uniformly defined as anti-inflammatory or pro-inflammatory. It ultimately depends on the subtypes and quantitative differences in these cells’ differentiation under various stimuli. Current research is ongoing, and future studies are expected to reveal this heterogeneity at a more microscopic level. For example, we may explore whether we can regulate the maturation and differentiation of meningeal B cells through skull bone marrow modulation.

On the other hand, aging and neurodegenerative diseases often appear as comorbidities, with aging itself being the biggest risk factor for neurodegenerative diseases [128]. The main source of B cells in the dura mater comes from the skull bone marrow under healthy conditions. However, with the aging of SMCs and fibrosis of the dura mater, the elimination of immune cells in the skull bone marrow is hindered, resulting in a decrease in B cells from the skull bone marrow in the dura mater and an increase in peripheral infiltration [74, 126, 129]. We speculate that this will have a certain impact on the occurrence and development of neurodegenerative diseases. Additionally, one particularly noteworthy point is that while the hematopoietic activity of bone marrow gradually decreases with age, skull bone marrow, unlike other bone marrow, remains hemodynamically active [25, 30, 130]. A recent study has identified unique expansion capabilities and anti-aging characteristics of skull bone marrow. Research indicates that skull bone marrow continues to expand throughout adulthood, accompanied by vascular growth and an increase in hematopoietic cells. Hematopoietic stem/progenitor cells (HSPCs) and the VEGFA signaling pathway play key roles in this process. Compared to long bone marrow, skull bone marrow exhibits greater adaptability, enabling rapid and dynamic adjustments during physiological changes (e.g., pregnancy) and pathological conditions (e.g., stroke and chronic myeloid leukemia). Remarkably, skull bone marrow demonstrates strong resistance to aging, effectively countering aging markers such as inflammation, adipogenesis, and loss of vascular integrity. CT scan analyses in humans further reveal that skull bone marrow volume significantly increases in elderly individuals, with this phenomenon being more pronounced in women, potentially due to gender-specific physiological and hormonal changes [56].

These findings suggest that the unique anti-aging properties of skull bone marrow may play a critical role in the onset and progression of neurodegenerative diseases. On one hand, the dynamic adaptability and anti-aging capacity of skull bone marrow may help delay the progression of neuroinflammation and immune dysregulation. On the other hand, age-related changes in skull bone marrow function may contribute to the pathological progression of neurodegenerative diseases by altering the immune environment of the dura mater. Future research should focus on further investigating the anti-aging mechanisms of skull bone marrow and its specific roles in neurodegenerative diseases, offering new directions for the development of immune regulation-based therapeutic strategies. For instance, therapeutic approaches could aim to improve dural immune balance by targeting the regulation of skull bone marrow hematopoietic activity or the VEGFA signaling pathway, thereby delaying the onset and progression of neurodegenerative diseases.

### Central nervous system tumors

CNS tumors are divided into primary nervous system tumors, such as glioblastoma (GBM), and metastatic nervous system tumors, particularly from the lung (20–56% of patients), breast (5–20%) and melanoma (7–16%) [131–134]. The prognosis of GBM is very poor, mainly due to the neglect of the relationship between tumor cells and the tumor microenvironment during the treatment process, in addition to the high degree of malignancy of the tumor itself [135–138]. Tumor-associated macrophages (TAMs), tumor-associated neutrophils (TANs), and myeloid-derived suppressor cells (MDSCs) are the major immune cell components in the GBM microenvironment [139–141]. However, whether TANs promote or inhibit tumors has always been controversial [140, 142, 143]. Recently, a study found that GBM promotes the migration of immature neutrophils from the skull bone marrow to the tumor microenvironment, where they differentiate into “hybrid” TANs with antigen-presenting capabilities under the influence of tumor-secreted factors such as GM-CSF and IFN-γ [144]. These TANs exhibit complex morphological and functional transcriptomic characteristics, enabling them to process antigens and activate CD8^+^ T cells through MHCII-dependent mechanisms, thereby exerting antitumor effects [144]. scRNA-seq has revealed the heterogeneity and differentiation trajectory of TAN, demonstrating their origin from local progenitor cells in the skull bone marrow rather than traditional circulating neutrophils [144]. Experimental evidence further identifies skull bone marrow as a significant source of myeloid antigen-presenting cells within the GBM immune microenvironment, with neutrophil migration driven by chemokines such as CXCL12 [144]. Moreover, studies show that the use of the CXCR4 inhibitor AMD3100 enhances the migration and antitumor function of skull bone marrow-derived neutrophils, leading to significant tumor volume reduction and prolonged survival in mouse models [144–146]. This study systematically highlights, for the first time, the critical role of skull bone marrow in shaping the GBM immune microenvironment and offers novel insights for developing immunotherapy strategies targeting skull bone marrow regulation.

The disease progression of peripheral cancer in the case of metastasis to leptomeninges is very fast, and the blow to patients is also devastating [147]. SMCs play a critical role in breast cancer meningeal metastasis by serving as a direct bridge between the skull bone marrow and the meninges. These channels provide tumor cells with an efficient and unconventional migration pathway that bypasses the BBB Studies have shown that breast cancer cells initially colonize the skull bone marrow, subsequently migrate along the outer membrane surface of emissary veins, and ultimately reach the meninges. Tumor cells exploit integrin α6 to bind to laminin in the channel’s basement membrane, enhancing their adhesion and migratory capabilities. Furthermore, the microenvironment of SMCs offers protection and support for tumor cells. Specifically, meningeal macrophages and other immune cells within the channels are hijacked by tumor-derived signals to secrete tumor-promoting factors, such as glial cell-derived neurotrophic factor (GDNF), which facilitate tumor survival, adaptation to the meningeal environment, and immune evasion. Experimental evidence indicates that the diameter of SMCs is significantly expanded in mouse models of meningeal metastasis. Moreover, blocking integrin α6 or inhibiting channel remodeling effectively reduces meningeal metastasis. These findings suggest that SMCs not only serve as a vital physical pathway for breast cancer meningeal metastasis but also promote tumor colonization and dissemination through their unique microenvironment, offering novel therapeutic targets for the treatment of meningeal metastasis [26, 148].

In vitro experiments on acute lymphoblastic leukemia, breast cancer, or lung cancer cells revealed that chemoattractants, which can promote the migration of malignant cells, were present in CSF [26, 149].

The critical role of skull bone marrow and its associated pathways in CNS tumors has been gradually revealed. It is not only an important source of immune cells but also profoundly influences tumor occurrence and development through complex migration mechanisms and microenvironmental regulation. Whether it is TANs in GBM or SMCs in breast cancer leptomeningeal metastases, these studies provide new targets and strategies for CNS tumor treatment. However, the translation from basic research to clinical application still faces challenges such as precise regulation of immune cell function, development of specific targeted drugs, and optimization of therapeutic adaptability. In the future, through in-depth research on the immune regulatory mechanisms of skull bone marrow combined with advanced technological approaches, it is expected to develop more efficient and safer treatment plans, bringing better prognosis and survival rates for CNS tumor patients.

## Conclusions and perspectives

The discovery of SMCs has opened a new avenue for our understanding of CNS immunity. It has not only drawn attention to skull bone marrow hematopoiesis but also challenged the traditional concept that infiltrating immune cells in the CNS are solely derived from peripheral bone marrow. The direct and bidirectional communication between CSF, containing various stimulating signals, and skull bone marrow allows the skull bone marrow to be the first to receive signals of changes in the CNS microenvironment. This prioritizes the role of skull bone marrow in the immune regulation of the CNS.

One notable exception is the TBI model. TBI is often accompanied by skull fractures and direct, severe damage to the BBB [86]. Additionally, the preparation of animal models for TBI is diverse. Here, we focus on TBI models prepared by CCI or FPI, because these require removing the mouse skull, which directly impacts the skull bone marrow hematopoietic niche and SMCs. The removal of the skull theoretically weakens the impact of skull bone marrow on the CNS after brain injury. This likely explains why there is only one unpublished study on TBI and skull bone marrow-related research before the writing of this review. The article points out that the number of cells derived from skull bone marrow is less than peripheral sources [55]. However, this view is one-sided because early removal of the skull significantly impacts the production and transportation of cells in the skull bone marrow. Considering the impact of skull bone marrow in other diseases, if a closed TBI model without removing the skull is chosen, the proportion of cell numbers may differ significantly.

The potential of skull bone marrow as a non-invasive diagnostic tool for neurological diseases has been increasingly acknowledged. Bone marrow puncture is a well-established method for extracting marrow cells [150], widely used in the diagnosis of hematological diseases. TSPO is a PET biomarker that is significantly upregulated during neuroinflammation. Unlike bone marrow puncture, these markers can be obtained through non-invasive PET scanning methods. In AD patients, TSPO-PET signals are significantly increased in the frontal, parietal, and motor cortices. In stroke and MS patients, the TSPO-PET signal is increased in the temporal pole, the skull base in relapsing-remitting MS and primary progressive MS patients, and in the frontal cortex and motor area in 4-repeat tauopathies. Similar results were obtained in AD animal models [39] (Table 3). Thus, the signal uptake of PET-TSPO by the skull is disease-specific, providing strong evidence for diagnosing different diseases and even comorbidities.

**Table 3:** Disease-specific signaling of TSPO-PET.

Disease	Distribution
AD	Frontal, parietal, motor
MS	Prefrontal, motor
Four-repeat tauopathies	Motor, temporal
Stroke	Temporal, skull base

During wartime, bone marrow injection served as an important alternative for fluid resuscitation when intravenous access was unavailable; however, it was limited to peripheral bone marrow sites, such as the sternum, humerus, and tibia [150]. In contrast, treating CNS diseases presents unique challenges due to the BBB. The BBB significantly limits the efficacy of CNS drugs, requiring repeated and high-dose peripheral administrations to achieve and maintain therapeutic levels in the brain [151, 152]. This approach often results in severe adverse reactions. Consequently, there is an urgent need for new drugs and delivery methods that can specifically target the CNS, enhance drug utilization in the brain, and reduce both dosage and administration frequency. Recent research has increasingly identified the skull as a promising therapeutic target. This approach offers the potential to bypass the BBB, providing significant advantages for treating CNS diseases with minimal systemic toxicity. Theodore L et al. influenced the neuroinflammatory response of TBI mice through transcranial administration [146], which also supports the existence of SMCs; Additionally, recent research found that immune cells gather around the sinus wall, with the largest group located at the rostral-rhinal confluence of the sinuses, which can be connected to the skull bone marrow through a DALT structure rostral-rhinal venolymphatic hub [153]. These immune cells can connect to the skull bone marrow through the DALT structure of the oral and nasal venous lymph nodes [153]. This also raises questions about whether the administration method through nasal inhalation is also related to the influence of immune cells in the bone marrow of the skull. Recent research indicates that intracalvariosseous administration (ICO), which involves applying drugs between the inner and outer layers of the skull, can increase drug concentrations in the brain by tens to hundreds of times compared to traditional intravenous administration [154]. This method, originally used when SMCs were first discovered, has been validated in AD mouse models, where intracranial administration significantly improves cognitive abilities. Additionally, ICO can effectively address compliance issues in patients with poor adherence, such as those with AD, through long-acting microspheres or pump infusion, thus promoting clinical translation [155]. The mouse model highlights the critical role of skull bone marrow and SMCs in the brain-immune axis. In contrast, human skull bone marrow anatomy is more complex, featuring wider and more prominent bone marrow cavities, more extensive connections with the meninges, and a more intricate SMC structure. Human SMCs not only allow immune cell migration but can also directly connect to arachnoid granulations, potentially participating in CSF sampling and energy metabolism. These unique anatomical, molecular, and functional characteristics suggest that human skull bone marrow and SMCs may play more significant roles and provide new perspectives and potential targets for the diagnosis, monitoring, and treatment of brain diseases. However, compared to peripheral bone surgery, skull surgery can cause patient hesitation due to potential complications like infection and bleeding, which can be fatal if not managed properly. Therefore, strict aseptic procedures and advanced surgical techniques are essential to minimize these risks. Despite these challenges, ICO offers an innovative and highly advantageous approach for precise CNS drug delivery, ultimately benefiting patients.

Thus, we can determine the lesion site based on the non-invasive diagnostic model of PET-TSPO, then combine it with transcranial or nasal administration to locally intervene at the lesion site, potentially reducing side effects caused by systemic drug application.

Of course, the discovery of SMCs and the emphasis on skull bone marrow hematopoiesis have only gone through about six years [25], and research on them is gradually improving.

However, there are still many questions that need to be explored (OPEN QUESTIONS). Recent studies have revealed that the CNS establishes dual connections with skull bone marrow and peripheral bone marrow through SMCs and the meningeal lymphatic system, respectively [25, 30, 129]. However, due to the lack of advanced technologies capable of dynamically monitoring immune cell migration and signal transmission in diverse immune environments, it remains unclear how the CNS coordinates interactions between skull bone marrow immunity and peripheral immunity under both healthy and pathological conditions. The diameter and density of SMCs vary across different regions of the skull [33] (Table 1). The causes of this heterogeneity and its potential effects on the migration and differentiation of immune cells originating from skull bone marrow remain unknown. Additionally, CSF connects the CNS with both skull and peripheral bone marrow through SMCs and the meningeal lymphatic system, respectively [20, 21, 30]. However, the absence of tools to selectively manipulate these two pathways has hindered efforts to quantify their relative contributions to immune regulation. Skull bone marrow has been found to possess unique expansion capacity and anti-aging characteristics [56]. With aging, the volume of the skull bone marrow cavity gradually increases, as does its contribution to systemic hematopoiesis [56]. Conversely, under certain pathological conditions, such as AD, the proportion of B cells originating from peripheral bone marrow infiltrating the dura mater increases with age [15, 30]. This raises the question: does SMC ossification create transport barriers for immune cells, including B cells? Trauma-induced damage to skull bone marrow and SMCs may disrupt their critical roles in immune regulation and CNS homeostasis. Skull bone marrow supplies immune cells to the CNS, while SMCs facilitate communication between skull bone marrow and the meninges. Damage to these structures may impair immune cell mobilization, prolong neuroinflammation, hinder tissue repair, and increase the risk of chronic inflammation, neurodegeneration, and systemic complications. Furthermore, damaged SMCs may compromise vascular integrity and BBB function, exacerbating long-term outcomes. As a critical medium linking central and systemic immunity, CSF may also mediate communication between immune cells from different sources [28, 33]. However, the extent to which this communication impacts overall immunity remains poorly understood. Existing experimental methods, such as bone marrow tagging using the microinjection technique, skull bone-flap transplantation, irradiation with partial shielding, photoconversion KikGR, and parabiosis, have provided valuable insights into skull bone marrow function [25, 30]. However, these methods are often invasive, technically challenging, and harmful to experimental animals, making them unsuitable for long-term, dynamic, and precise studies of the skull bone marrow immune system.

To address these challenges, there is an urgent need to develop less invasive and more accessible labeling techniques that minimize harm to experimental animals. Combining these approaches with cutting-edge multidisciplinary technologies, such as high-resolution imaging and scRNA-seq, could enable real-time tracking and functional localization of cells from different sources. These advancements will not only resolve existing scientific gaps but also provide critical insights into the diagnosis and treatment of various CNS diseases. Research on skull bone marrow and SMCs is still in its infancy. However, even solving small scientific questions in this field could have significant implications for the development of treatments for neurological diseases.
